# Use of Caffeine and Inducing Parturition with Dual Administration of Prostaglandin F2α in Gilts and Its Effect on Neonatal Vitality and Performance at Birth

**DOI:** 10.3390/ani15070984

**Published:** 2025-03-29

**Authors:** Adrián Alejandro Corrales-Hernández, Herlinda Bonilla-Jaime, Héctor O. Orozco-Gregorio, Ofelia Limón-Morales, Luis Alberto de la Cruz-Cruz, Elías Chávez-Delgadillo, Patricia Roldán-Santiago

**Affiliations:** 1 Programa de Doctorado en Ciencias de la Producción y de la Salud Animal, Universidad Nacional Autónoma de México, Avenida Universidad, Ciudad de México 04510, Mexico; adralexch@gmail.com; 2 Departamento de Biología de la Reproducción, Universidad Autónoma Metropolitana, Unidad Iztapalapa, Ciudad de México 09340, Mexico; lindabon35@gmail.com (H.B.-J.); ofelia.limon@yahoo.com (O.L.-M.); 3 Ingenieria en Producción Animal, Universidad Politécnica de Francisco I. Madero, Domicilio Conocido s/n, Tepatepec, Francisco I. Madero 42660, Mexico; gohector72@yahoo.com.mx; 4 Producción Agrícola y Animal, Universidad Autónoma Metropolitana, Calzada del Hueso 1100, Coapa, Villa Quietud, Coyoacán, Ciudad de México 04960, Mexico; ladelacruzcc@gmail.com; 5 Agropecuaria FC S.A. de C.V. Antiguo Camino a San Agustín #3, Ampliación Cadena Maquixco, Teotihuacán Estado de México 55843, Mexico; elias@forrajeselcorral.com; 6 Departamento de Reproducción, Facultad de Medicina Veterinaria y Zootecnia, Universidad Nacional Autónoma de México, Avenida Universidad, Ciudad de México 04510, Mexico

**Keywords:** gilt, neonate, caffeine, PGF2α, vitality, thermography, oximetry

## Abstract

One of the principal problems in swine production is neonatal mortality, especially in primiparous sows. Caffeine has been shown to be an efficacious neonatal stimulant that reduces mortality while improving key metabolic indicators associated with hypoxic processes. The aim of this study was to evaluate the effect of inducing parturition with a full dose (FD) (175 μg) or split dose (SD) (87.5 + 87.5 μg) of cloprostenol (PGF2α), with and without the administration of a single dose of caffeine (420 mg), on the vitality, clinical status, surface temperature, and weight at weaning of neonate pigs. In this experiment, the duration of farrowing was affected by the induction technique, as the PGF2α SD and PGF2α SD + caffeine treatment groups reduced this time by as much as 100 min compared to PGF2α FD and PGF2α FD + caffeine. The groups that received caffeine presented the best values for the indicators of the neonate performance, peripheral oxygen saturation (SpO2), glucose, surface temperature at 24 h, and weight at 21 days, especially the PGF2α SD + caffeine group. PGF2α FD had the lowest neonate performance and metabolic variables. Inducing birth with PGF2α SD is more efficient, and adding caffeine improves the performance and metabolic variables of piglets born from primiparous sows.

## Simple Summary

Currently, one of the problems that most affects swine production is neonatal mortality, a problem that is more intense in gilts. Seeking to implement treatments applicable in swine production at a commercial level, caffeine has been selected as a neonatal stimulant to reduce piglet mortality. However, one of the most common practices in swine production that cannot be ignored is the induction of parturition. Therefore, caffeine has been combined with two induction techniques, the full dosing of prostaglandins F2alpha and the same dose divided into two applications. The split-dose induction technique reduced the duration of farrowing by 100 min while the application of caffeine positively affected the piglets which presented the best values for the indicators of the neonate performance, metabolic variables, the surface temperature at 24 h, and weight at 21 days. Our experiment’s results showed that using caffeine with PGF2alpha to induce farrowing, combined with the split-dose technique, improves the performance and metabolic variables of piglets born to gilts, which would contribute to increased survival as well as animal welfare in farm.

## 1. Introduction

Neonatal mortality due to perinatal asphyxia is one of the main economic problems and risk factors for animal welfare in swine production, as it affects 16–20% of the piglets born per litter. It is also one of the principal causes of stillbirths and is involved in crushing and processes of starvation [1]. This problem occurs more often in gilts because they are still in the stage of growth and development and have not reached their maximum size [2]. This means they must distribute their energy and the nutrients they consume among their own requirements for maintenance, growth, fetoplacental development, and lactation [3,4,5,6]. In this regard, studies have reported that the piglets of primiparous sows may present up to 8% lower birth weights [7,8], lower rectal temperatures (up to 1 °C) [9], up to 0.9 °C lower surface temperatures [10], and reduced colostrum consumption by 50 g [11], and show 30 g less daily body weight gain in the first 21 days postpartum [9,12]. Moreover, these piglets present lower vitality than neonates born to multiparous sows [8].

In response to this, studies are underway to develop strategies that will help neonates born to gilts respond better to the complications that may occur during parturition, associated with processes of intrapartum hypoxia [13]. It is also important to consider that a common practice on swine farms is to induce births using PGF2α or its synthetic analog, cloprostenol, to program the activities of their trained personnel and ensure they will be present to efficiently supervise dams and their offspring during birth and thus help increase neonate survival. There are two techniques for inducing birth with prostaglandins. The first involves administering a single complete intramuscular dose (IM), while in the other, the dose is split into two injections, the second one applied 6 h after the first to reduce variation in the time within which parturition begins and improve the scheduling of birth events [14].

A strategy used to directly increase neonatal survival consists in administering caffeine, a substance that has been utilized as a neonatal stimulant since the decade of 2010 with satisfactory results in swine neonates that suffer hypoxic processes at birth [15]. Earlier studies reported that caffeine facilitates re-establishing the metabolic profile of hypoxic neonates 24 h after administration [16] and improves their thermoregulating capacity [17,18,19,20], two measures that increase vitality [19]. Caffeine acts by stimulating the respiratory power and neuronal contractibility and increasing concentrations of cyclic adenosine monophosphate (cAMP) [21]. To date, however, caffeine has only been tested in isolation, so its potential effects on treating intrapartum asphyxia in piglets when administered in combination with the induced parturition of sows are unknown. Therefore, the objective of the present study was to evaluate the effect of administering caffeine to gestating gilts induced with a full, or divided, dose of prostaglandins on the vitality, clinical status, surface temperature, and weight at weaning of their neonates.

## 2. Materials and Methods

The study was conducted on an experimental farm in the northeastern area of the State of Mexico where the climate is temperate to semi-cold and subhumid. The farm keeps an average of 100 sows. The study protocol was approved by the Institutional Committee for the Care and Use of Experimental Animals (CICUAE) of the Universidad Nacional Autónoma de México (UNAM) under protocol no. SICUAE.SD-2021/3-1.

### 2.1. Installations and Environment

The sows were housed in groups in farrowing corrals with concrete floors. They had ad libitum access to water and were fed 2.5–3.0 kg/day of a gestation diet according to their nutritional requirements [22]. Later, during lactation, the diet was changed to one based on corn–soya until day 21 postpartum.

One week before the probable parturition date, the dams were moved to the farm’s maternity area where an average minimum temperature of 18.9 °C and a maximum temperature of 26.9 °C were maintained with relative humidity in the range of 46.4–73.4%. They were housed individually in small pens with plastic slats where floors measured length area 1.8 × 2.4 m inside the pen, and the sows stayed in a cage made of conventional steel tubes that measured 0.6 × 2.1 m, each one equipped with a plastic eating bowl and 2 drinking bottles with metallic dispensers to ensure the uninterrupted availability of water. One was placed next to the dam’s feeding bowl, the other—20 cm high—for her piglets. The area for the piglets had a 0.37 × 1.2 m electric mat covered with a plastic pad that maintained an average temperature of 38.5 °C.

### 2.2. Animals and Treatments

**Figure 1:**
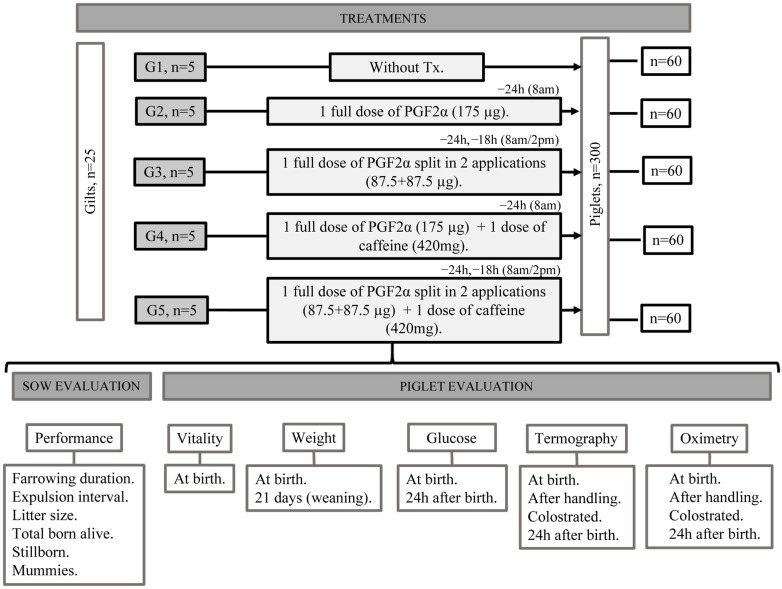
Description of the parameters evaluated and analyzed in gilts and piglets. G: Group, Tx: Treatment, PGF2α: Prostaglandin F2α.

A total of 25 nulliparous, York–Landrace sows and their litters were studied. This study included the first 12 piglets born per sow, regardless of litter size (300 piglets in all). The dams were distributed in 5 study groups according to the treatment they would receive before the onset of parturition: G1: control; G2: sows induced with a full dose of prostaglandins (cloprostenol 175 μg) (PGF2α FD); G3: sows induced with the split dose of prostaglandins (cloprostenol 87.5 + 87.5 μg) (PGF2α SD); G4: sows induced with a full dose of prostaglandins plus the administration of caffeine (cloprostenol 175 μg + 420 mg caffeine) (PGF2α FD + caffeine); and G5: sows induced with the split dose of prostaglandins (cloprostenol 87.5 + 87.5 μg) plus caffeine (420 mg) (PGF2α SD + caffeine) (Figure 1). Parturition was synchronized using cloprostenol sodium (Celoprost^®^, ParFarm Laboratorios de productos Veterinarios, MX, México City, Mexico) at 175 μg IM 24 h before the probable farrowing date, considering an average of 115 days of gestation for the sows that received the full dose (175 μg in one administration applied at 8:00 h, 24 h before the expected date of parturition) and for the ones treated with the split dose (1 dose of 175 μg divided into 2 applications of 87.5 μg each, the first administered 24 h before the expected parturition date, the second 6 h later, at 8:00 and 14:00 h). This protocol followed the methodology of Decaluwe et al. [14], who analyzed administering PGF2α split into two doses. The caffeine used was caffeine anhydrous (Sigma-Aldrich, Caffeine powder, ReagentPlus^®^ (CAS-No 58-08-02), Wuhan, China). The dose of caffeine administered intravaginally to the G4 and G5 sows was determined based on previous studies by Orozco-Gregorio et al. [16] and Sánchez-Salcedo et al. [19], and was set at 420 mg.

### 2.3. Sow Performance

The following reproductive variables were recorded for the sows: duration of farrowing (time between the first and last piglet born), total number of piglets per litter, number of liveborn piglets per litter, and number of stillbirths and mummified fetuses at birth. In addition, this study considered the expulsion interval, registered as the time between the birth of one piglet and the next.

### 2.4. Neonate Vitality

The evaluation of the vitality of the piglets began immediately after birth using the vitality indicators described by Orozco-Gregorio, et al. [23]. The parameters assessed were as follows: The heart rate (HR) (beats per minute, bpm), measured with a pulse-oximeter (Edan Instruments Inc., Nanshan, Shenzhen, China). Latency to first respiration is defined as the time between expulsion and the piglet’s first attempt to inspire air on its own. The coloration of the skin of the snout is based on observation and categorized as pale, cyanotic, or pink. Latency to standing is measured as the time between expulsion and the moment that the piglet could stand on its four extremities. Finally, the presence of meconium staining on the skin is classified as severe when it covers over 50% of the piglet’s body surface. It was moderate when it covered less than 50% of the body surface. It was absent when there was no evidence of meconium staining. The umbilical cord was also evaluated as an adhered and ruptured cord.

### 2.5. Blood Sampling

Two blood samples were drawn from each piglet: one at birth, the second at 24 h of extrauterine life. The first sample was taken using the technique described by Westgate, et al. [24]; that is, by drawing blood directly from the umbilical artery using previously heparinized syringes (Inhepar^®^ 1000 UI/mL, México City, Mexico) to prevent rapid coagulation. Later, blood glucose concentrations were measured with one drop of the sample in an Accu-Chek^®^ (Corydon, IN, USA), Performance device that gave instant readings. The results were recorded individually in accordance with the methodology by Nuntapaitoon and Tummaruk [25].

The second sample was drawn at 24 h of extrauterine life. The procedure entailed locating the external jugular vein by accurately defining the jugular groove after gently constraining the piglet. Each neonate was placed in a sternal recumbency position with its posterior limbs placed in the interior area of the arms and each anterior limb immobilized manually. To take the sample, the piglet’s head was held still by applying pressure to the jaw with the thumb and, at the same time, using light force to immobilize its neck. This technique helped define the jugular groove, the correct zone for drawing the sample. Later, one drop of this blood was tested with the Accu-Chek^®^ Performance device (Corydon, IN, USA). We should emphasize that to avoid altering the blood variables, all blood samples were taken in less than 30 s, counted from the moment the piglet was constrained to when the total sample was obtained [26]. Both samples were drawn by two people trained in constraining piglets and extracting blood.

### 2.6. Thermography

Thermographic images were taken at a distance < 20 cm with an HTI^®^ (Dongguan, China) model H18 camera with emissivity set at 0.95. Images were assessed at four moments: at birth (5 min postpartum); after the farm’s initial handling routine (drying the piglet and cutting the umbilical cord); after receiving colostrum; and at 24 h of age. Images were obtained from the area around the palpebral posterior medial margin of the lower eyelid (lacrimal caruncle) on the right side, as this is considered a zone that accurately reflects the animal’s internal temperature [27], and from the anterior region of the base of the ear [28]. Once all the thermographic images were obtained, they were classified to perform a follow-up on each neonate. All images were analyzed with IR ImageTools software (V1.0.0.13) and used to calculate mean temperatures.

### 2.7. Oximetry

Pulse-oximetry is a simple, non-invasive technique that measures oxygen saturation [29]. The pulse-oximeter for veterinary use (Edan Instruments Inc., Nanshan, Shenzhen, China) employed was equipped with a neonatal sensor to measure the peripheral oxygen saturation (SpO2%) and it was placed on the medial area of the piglet’s right ear after cleaning the residue of the fetal membranes, following the methodology by Vongsariyavanich, et al. [30]. This variable was also measured at four moments: at birth (5 min post-expulsion); after initial handling; after receiving colostrum; and at 24 h of age.

### 2.8. Weight

The body weight of the neonates was measured at birth (after drying but before receiving colostrum) and at 21 days of age. To measure the birth weight, the piglets were held by their front limbs at the hock area and weighed on a portable digital scale (Minicrane model MNCS-M, Shiyan, China). At 21 days of age, they were weighed individually on an electric platform scale (TCS-60 Platform Scale, Jinzhong, China). Each piglet was identified with a tattoo on one ear to facilitate follow-up on their weight on day 21.

### 2.9. Analysis Statistical

All statistical analyses were carried out with JMP 8.0 (JMP Institute, Marlow, Buckinghamshire, UK). All data were explored to determine distributions using the JMP univariate procedure and normality tests were run for all variables. Those that did not achieve normality were transformed to a logarithmic scale and presented as mean least squares (GLIMMIX).

The piglet and the dam, respectively, were set as the random and fixed effect in a model of analysis of variance (ANOVA) for all variables.

Continuous data were organized as mean ± SD and compared between groups by means of the ANOVA following the GLM procedure (General Linear Models). When significant between-group differences were found, a Tukey Kramer multiple comparison of means test was conducted with the level of significance set at *p* < 0.05. All data are presented as mean ± SEM. Significant differences of means between groups were determined at *p* < 0.05. All differences between the groups and in the vitality indicators were analyzed using a χ^2^ test. Confidence intervals (CI) were calculated at the 95% level.

## 3. Results

Table 1 presents the results of the reproductive performance of the sows during parturition in relation to the effect of the different treatments evaluated. Significant differences were found in the duration of parturition (*p* = 0.0329), as the PGF2α SD and PGF2α SD + caffeine groups presented the lowest birth times, while PGF2α FD + caffeine and PGF2α FD had birth times of 37 and 39 min, respectively, longer than the control group. For the between-birth intervals, PGF2α SD and PGF2α SD + caffeine also showed lower times than the control group (*p* = 0.0204).

The litter size averaged at 12.8–14.0 total piglets. The groups with the lowest number of stillborn piglets were PGF2α FD + caffeine, PGF2α SD, and PGF2α SD + caffeine (*p* < 0.0001), while PGF2α FD had the most (*p* < 0.0001). The presence of mummified fetuses was higher in PGF2α FD and PGF2α SD + caffeine than in the other groups (*p* < 0.0001).

Table 2 shows the indicators of the neonate’s performance at birth. The piglets born to sows that received the PGF2α SD + caffeine, PGF2α FD + caffeine, and PGF2α SD treatments do not present significant differences and had the highest HR. In contrast, the control and PGF2α FD groups presented the lowest HR values of all the study groups (*p* < 0.0001).

In terms of latency to first respiration, the group that took the longest was PGF2α SD + caffeine (*p* ≤ 0.0001). No significant differences were found among the other groups. Regarding the time required to stand up, PGF2α FD took longer to perform this action than PGF2α FD + caffeine and PGF2α SD + caffeine (*p* = 0.0014). For latency to taking the teat, PGF2α SD + caffeine and PGF2α FD + caffeine had the shortest times (*p* < 0.0001). No significant differences were found in the other groups.

Table 3 displays the vitality indicators of the newborn piglets from the five study groups. Most groups presented high percentages of piglets born by cranial presentation, but PGF2α SD and the control group had the highest values (*p* < 0.0001), while for caudal presentations, the groups with higher percentages were PGF2α SD + caffeine and PGF2α FD (*p* < 0.0001). For the variable meconium staining, the control and PGF2α SD groups had the highest percentages of the absence of staining (*p* < 0.0001), while the group with the greatest presence of the moderate condition was PGF2α FD + caffeine (*p* < 0.0001). One group, PGF2α FD, had severe staining (*p* < 0.0001). With respect to the variable color at birth, four groups presented scores of 100% for a pink color; the exception was PGF2α FD + caffeine, which had scores of 91.66% for a pink color (*p* < 0.0001) and 8.34% for pale piglets. Finally, for the variable umbilical cord, the highest percentages of adhered cords were seen in the control and PGF2α FD + caffeine groups at 90 and 87.12%, respectively (*p* < 0.0001). PGF2α FD had the highest percentage of ruptured cords at 24.49% (*p* < 0.0001).

Table 4 presents the results of the clinical indicators evaluated in the neonates (1) at birth; (2) immediately after initial handling (cutting the umbilical cord and drying); (3) after receiving colostrum; and (4) at 24 h postpartum. For the indicator SpO2 (%), upon expulsion, the piglets in PGF2α FD + caffeine had the lowest values of all groups (*p* < 0.0001). No significant differences were observed among the others. Likewise, no significant between-group differences were found for the variable handling. After the piglets received colostrum, we observed a significant difference only between the control and PGF2α SD + caffeine groups compared to PGF2α SD. Later, at the 24 h mark, PGF2α FD + caffeine and PGF2α SD + caffeine had higher values than the control and PGF2α SD groups (*p* ≤ 0.0001). Finally, the values for PGF2α FD were also significantly higher than those of the control group (*p* ≤ 0.0001).

Turning to blood glucose levels (mg/dL), the values recorded at birth and at 24 h of age are shown. The former showed that only PGF2α SD and PGF2α SD + caffeine had significantly lower values (*p* < 0.0001) than the control group and the other two experimental groups. In contrast, at 24 h, no significant differences were found among the experimental groups, though all four showed glucose values significantly higher (approximately 11%, *p* = 0.0007) than the control group.

Table 5 presents the average temperatures of the lacrimal caruncles of the piglets in the study groups at birth, after initial handling (see above), after receiving colostrum, and at 24 h postpartum. At birth and after drying, the PGF2α FD piglets had the highest temperature of all groups. After receiving colostrum, the control and PGF2α FD groups presented higher temperatures than the others (*p* < 0.0001). At 24 h, PGF2α FD had a higher average temperature than the other groups (*p* < 0.0001).

Table 6 displays the average temperatures measured at the anterior base of the ear of the piglets of the primiparous dams. At birth, the values for group PGF2α FD were higher (*p* < 0.0001) than those of the other groups. No significant differences were found among the other groups.

For the post-initial handling stage, the temperature values for PGF2α SD, PGF2α FD + caffeine, and PGF2α SD + caffeine did not present significant differences, but all three had values significantly lower than those of the control and PGF2α FD groups (*p* < 0.0001). After the piglets received colostrum, the values for the control and PGF2α FD groups were similar, and both were significantly higher than those of the other groups (*p* < 0.0001). In the evaluation conducted at 24 h, the PGF2α SD + caffeine group presented the highest temperature of all the study groups, though its values were similar to those of PGF2α FD + caffeine and PGF2α FD (*p* < 0.0001).

Table 7 shows the weight (in grams) of the piglets of each group evaluated at birth and at 21 days of life (weaning). Regarding birth weights at day 0, we only observed significant differences between the group PGF2α FD + caffeine and the control and PGF2α SD groups. For the piglets’ weight at day 21, the values for PGF2α SD + caffeine were significantly higher than those of the control, PGF2α FD, and PGF2α SD groups (*p* < 0.0001).

## 4. Discussion

Genetic improvements in modern production have increased the litter size [31] to as many as 23 piglets [32], but this has prolonged the duration of farrowing and increased the probability that some neonates will suffer intrapartum hypoxia, with the consequent loss of vitality, a reduced thermoregulating capacity, the possible appearance of metabolic alterations [33], and greater competition to reach the mother’s udder [34]. In the present study, the duration of birth was influenced by the induction method, but not by caffeine administration [20]. Farrowing times in the PGF2α SD and PGF2α SD + caffeine treatment groups decreased by as much as 100 min compared to PGF2α FD and PGF2α FD + caffeine. These results coincide with those of Tospitakkul, et al. [11], where the authors found lower farrowing durations with the SD technique compared to induction with an FD, with differences as large as 50 min (*p* < 0.05). Likewise, the shortest between-expulsion intervals were observed in PGF2α SD and PGF2α SD + caffeine, suggesting that the induction method impacted these results, as the divided dose extended the time during which cloprostenol maintained its stimulant effect due to its half-life of up to 3 h [11,14].

Overall, the piglets in the groups that received caffeine presented better values for the vitality and performance indicators. Moreover, if we compare the induction method, the piglets born in study groups PGF2α SD and PGF2α SD + caffeine had a better performance than the ones in PGF2α FD and PGF2α FD + caffeine in terms of the latencies to standing and taking the teat, and their HR values. These findings could be related to the stimulation of the central nervous system, specifically, enhanced alert states and reduced fatigue due to caffeine’s effect on competitively inhibiting the A1 and A2A adenosine receptors that increase neuronal activation [21] and improve the neonates’ capacity to move and adapt to extrauterine life [35]. The PGF2α FD group, in contrast, achieved the lowest scores for the vitality and performance indicators, as a higher percentage of piglets had moderate meconium staining and torn umbilical cords, two parameters that may have been affected by the prolonged expulsion times [36,37] that PGF2α FD presented [11].

With respect to the indicator oxygen saturation, at birth, the PGF2α FD + caffeine piglets had the lowest levels, likely due to their prolonged birth times [36] associated with the FD induction technique [11]. However, these values were re-established at 24 h of life, a rapid recovery possibly influenced by the effects of caffeine on the respiratory impulse due to the antagonism of the A1 adenosine receptors in the pons and medulla oblongata [38] that enhances the sensitivity of the chemoreceptors to CO2 [39], and to the blocking of the A2A adenosine receptors, which improves the oxygen consumption [40] and cardiac output [41].

For blood glucose levels, PGF2α SD and PGF2α SD + caffeine were the only groups that achieved values within normal parameters at birth, as the others had high levels [42]. This result could be related to the SD induction technique that lowered the duration of parturition compared to the groups induced by the FD technique, such that the piglets presented fewer cumulative effects of birth order and initial body weight [36], though it may also involve the characteristics of these primiparous sows [8]. The high glucose levels in the neonates of the PGF2α FD and PGF2α FD + caffeine groups could be a consequence of suffering during parturition since the stress generated by the birth process releases adrenaline and noradrenaline [43], two hormones that activate glycogenolysis, thus increasing glucose [36,44]. In addition, Panzardi, et al. [42] mentions that both high and low glucose levels in swine are indicators of a low survival ability during the first week of life. At 24 h, the control group presented the lowest glucose values. In this regard, the values of the groups that received caffeine could be related to both this substance’s stimulating effect on the metabolic rate and the induction method. Caffeine competitively inhibits the A1 and A2A receptors, so it increases circulating catecholamines [45], cortisol, and glucagon, all of which foster the glycogenolysis process that hydrolyzes glycogen reserves in the liver and muscles to transform them into glucose, thus raising the plasma levels [46].

But caffeine also reduces sensitivity to insulin [47,48], thus generating resistance that impedes glucose from entering the cells, so concentrations increase in the blood. Birth times were longer for the groups induced with FD, so this finding may be associated with greater stress in the piglets and the resulting increase in glucose, perhaps attributable to a compensatory effect in response to a state of fetal suffering during birth [43,49].

The piglets born to the sows treated with PGF2α SD also experienced shorter birth periods, a condition that may have contributed to their better scores on the performance indicators at birth [50], especially for taking the udder, which allowed them to consume colostrum more efficaciously. Added to this, the PGF2α SD + caffeine treatment may have conferred an even greater advantage due to the stimulating effect of caffeine, which was reflected upon comparing the values of the control group. These results are similar to those in Superchi et al. [17] and Sánchez-Salcedo et al. [19], where the groups treated with caffeine showed enhanced viability. It is also important to consider that the piglets born to primiparous sows tend to show a higher proportion of energy imbalances compared to those born to sows with two to six parities [8].

In general, the piglets in PGF2a FD presented the highest temperatures in both the lacrimal caruncle and the base of the ear at birth, after initial handling, and after receiving colostrum. This could be related to the higher amount of glucose released during parturition and, hence, to an increased postpartum metabolism, attributable to a compensatory effect for the suffering experienced during birth [42,43] caused by the duration of expulsion.

At birth, the piglets in PGF2α FD + caffeine and PGF2α SD + caffeine reflected the stimulating effects of caffeine by showing greater vigor, as they stood up faster [19] and increased their locomotor activity [51] to seek the teat and consume colostrum more efficiently [17]. This may have contributed to their improved thermoregulating capacity as well [52,53]. These groups also presented a higher average surface temperature at 24 h, possibly because supplementing the sows with caffeine improved this ability [17,18,19,54]. Caffeine increases both circulating catecholamines [45] and cortisol, which stimulate gluconeogenesis to produce glucose from alternative precursors [55], while also favoring temperature increases due to the faster metabolic rate. This could explain the temperatures at 24 h in the PGF2α FD + caffeine and PGF2α SD + caffeine groups. At 24 h postpartum, these two groups had higher surface temperatures than the control and PGF2α SD groups in the lacrimal caruncle (at least 0.4 °C) and at the base of the ear (up to 0.9 °C higher). In this regard, Roldán-Santiago et al. [10] mentions that piglets with low vitality have lower temperatures than those with higher vitality, with differences that can be as large as 0.71 °C. In our study, the PGF2a FD piglets had the lowest scores for most of the performance and vitality indicators, and their temperatures coincided with the group of piglets with low vitality, according to Roldán-Santiago et al. [8].

It is unlikely that the differences recorded in the initial weight of the piglets resulted from administering caffeine, since it was applied on day 114 of gestation when the fetuses were already physically developed. Moreover, the amount applied was low (a single dose of 0.420 mg, 1 day prior to parturition) compared to those used in earlier studies, as in two studies that recorded this effect: Superchi et al. [17] (4.8 g per day for three days prior to birth) and Dearlove et al. [18] (a single dose of 6.075 g one day before farrowing). Likewise, Tospitakkul et al. [11] established that the SD and FD induction techniques do not affect birth weights when applied correctly. The weights of the piglets on day 21 were, however, influenced by the treatments applied, as PGF2α SD + caffeine presented the highest daily weight gain, followed by PGF2α FD + caffeine (4866 g), likely due to their increased vitality [16,19] due to the effect of caffeine [56], which allowed them to compete for colostrum more efficaciously and gain an initial advantage. Other effects may also be involved, such as the birth weight, colostrum production by the dam (which is lower in primiparous sows), and the litter size.

## 5. Conclusions

Our results suggest that administering caffeine to gestating primiparous sows and inducing birth with a full or split dose of prostaglandin had positive effects on the vitality, clinical status, surface temperature, and weight at weaning of swine neonates. With respect to their vitality, the piglets in the groups that received caffeine stood up more quickly and were able to take the teat faster than those in the other groups, two indications of a faster adaptation to the extrauterine environment. Regarding the surface temperature, the groups treated with caffeine presented values 0.4–0.9 °C higher than the others. In the case of weight, the PGF2α SD + caffeine piglets achieved greater daily weight gain than those in the other groups, in a range of 15–30 g. Overall, the group of sows and their litters that presented the best performance at birth was the one treated with the combination of PGF2α in the split dose supplemented with caffeine. Therefore, we can affirm that applying caffeine as a prophylactic treatment confers a great advantage by providing immediate protection to neonates that might suffer perinatal hypoxia. In addition, this treatment can help piglets adapt more adequately to extrauterine life by increasing their vigor and improving their thermoregulatory and respiratory capacity when used in combination with the divided dose induction technique.

## Figures and Tables

**Table 1:** Performance indicators of gilts induced with PGF2α using split-dose and full-dose technique with and without caffeine administration.

	Control (n = 5 Gilts)	PGF2α FD (n = 5 Gilts)	PGF2α SD (n = 5 Gilts)	PGF2α FD+ Caffeine (n = 5 Gilts)	PGF2α SD + Caffeine (n = 5 Gilts)	*p*-Value
	Mean ± SE	Mean ± SE	Mean ± SE	Mean ± SE	Mean ± SE	
Farrowing duration (min)	200.40 ± 30.72 AB	239.40 ± 30.72 A	122.40 ± 30.72 B	237.80 ± 30.72 A	136.00 ± 30.72 B	0.0329 *
Expulsion interval (min)	14.02 ± 2.13 B	19.68 ± 2.89 A	12.89 ± 1.97 B	15.57 ± 2.26 AB	9.65 ± 2.13 B	0.0204 *
Total number of piglets born per litter TB (n)	12.8 ± 0.7 BC	14.0 ± 0.5 A	13.6 ± 0.4 B	13.4 ± 0.3 B	13.6 ± 0.2 BC	0.7470
Number of piglets born alive per litter (n)	11.8 ± 0.5 A	12.8 ± 0.6 A	13.0 ± 0.4 A	13.0 ± 0.71 A	13.00 ± 0.9 A	0.7470
Number of stillborn (n)	0.89 ± 0.1 A	0.99 ± 0.19 A	0.69 ± 0.15 AB	0.42 ± 0.15 B	0.49 ± 0.1 B	<0.0001 *
Number of mummies per litter (n)	0.21 ± 0.01 B	0.25 ± 0.1 A	0.12 ± 0.4 C	0 ± 0.0 D	0.24 ± 0.1 A	<0.0001 *

Data presented as mean standard error. *p* > 0.05. n = number of piglets. A, B, C, D, Different superscript letters within a row denote data that differ significantly, Tukey (*p* < 0.05). PGF2α, Prostaglandin F2alpha; FD, full dose; SD, split dose; SE, Standard Error; TB, total number of piglets born. * Denotes data that differ significantly.

**Table 2:** Performance indicators of neonates (n = 300) born from gilts induced with PGF2α using split-dose and full-dose technique with and without caffeine administration.

	Control	PGF2α FD	PGF2α SD	PGF2α FD + Caffeine	PGF2α SD + Caffeine	*p*-Value
	Mean ± SE	Mean ± SE	Mean ± SE	Mean ± SE	Mean ± SE	
Heart rate (bpm)	135.10 ± 3.03 B	142.20 ± 3.06 B	155.27 ± 2.20 A	158.24 ± 3.52 A	164.37 ± 3.39 A	<0.0001 *
Latency to first respiration (s)	5.52 ± 0.33 B	5.59 ± 0.34 B	4.51 ± 0.24 B	5.34 ± 0.38 B	7.14 ± 0.37 A	<0.0001 *
Latency to stand up (s)	116.78 ± 6.62 AB	133.42 ± 6.43 A	124.64 ± 7.41 AB	98.03 ± 6.47 B	102.08 ± 7.92 B	0.0014 *
Latency to connecting to the dam’s teat (min)	23.64 ± 1.30 A	18.97 ± 1.32 A	18.76 ± 0.95 A	15.05 ± 1.48 B	14.90 ± 1.44 B	<0.0001 *

A, B, Different superscript letters within a row denote data that differ significantly, Tukey (*p* < 0.05). PGF2α, Prostaglandin F2alpha; FD, full dose; SD, split dose; SE, Standard Error; bpm, beats per minute; s, Seconds; min, Minutes. * Denotes data that differ significantly.

**Table 3:** Vitality indicators of neonatal piglets (n = 300) born from gilts induced with PGF2α using split-dose and full-dose technique with and without caffeine administration.

		Control	PGF2α FD	PGF2α SD	PGF2α FD + Caffeine	PGF2α SD + Caffeine	*p*-Value
Presentation (%)							
	Cranial	78.44 *	77.55	81.05 *	72.98	71.42	<0.0001 *
	Caudal	21.56	22.45 *	18.95	27.02	28.58 *	<0.0001 *
Meconium staining (%)							
	Absent	78 *	51.86	60.64 *	36.12	61.76	<0.0001 *
	Moderate	20	22.22	22.34	61.11 *	26.2	<0.0001 *
	Severe	2	25.92 *	17.02	2.77	12.04 *	<0.0001 *
Skin color (%)							
	Pink	100	100	100	91.66 *	100	<0.0001 *
	Pale	0	0	0	8.34	0	NA
	Cyanotic	0	0	0	0	0	NA
Umbilical cord condition (%)							
	Adhered	90 *	75.51	88.03	87.12 *	87.34	<0.0001 *
	Rupture	10	24.49 *	11.97	12.88	12.66	<0.0001 *

Percentage of total piglets born in each study group. Chi-Square (*p* < 0.05). * Denotes data that differ significantly. PGF2α, Prostaglandin F2alpha; FD, full dose; SD, split dose.

**Table 4:** Effect of induction of parturition and administration of caffeine in gilts on SpO2 (%) and glucose (mg/dL) over their piglets (n = 300).

Variable	Time	Control	PGF2α FD	PGF2α SD	PGF2α FD + Caffeine	PGF2α SD + Caffeine	*p*-Value
		Mean ± SE	Mean ± SE	Mean ± SE	Mean ± SE	Mean ± SE	
SpO_2_ (%)	At birth	84.74 ± 1.32 A	80.85 ± 1.37 A	82.31 ± 0.97 A	74.72 ± 1.54 B	85.02 ± 1.50 A	<0.0001 *
	After handling	82.28 ± 1.54 A	79.95 ± 1.64 A	79.36 ± 1.14 A	83.00 ± 1.81 A	84.66 ± 1.74 A	0.077
	Colostrated	89.36 ± 0.94 A	85.93 ± 0.97 AB	85.45 ± 0.71 B	87.54 ± 1.12 AB	89.87 ± 1.06 A	0.0009 *
	24 h after birth	84.76 ± 0.89 C	88.34 ± 0.92 AB	86.58 ± 0.66 BC	91.10 ± 1.04 A	91.87 ± 1.01 A	<0.0001 *
Glucose (mg/dL)	At birth	52.51 ± 1.66 A	58.85 ± 1.79 A	42.98 ± 1.29 B	53.07 ± 1.86 A	42.55 ± 1.94 B	<0.0001 *
	24 h after birth	99.24 ± 2.43 B	113.62 ± 2.71 A	107.76 ± 1.91 A	111.56 ± 2.75 A	110.84 ± 2.78 A	0.0007 *

A, B, C Different superscript letters within a row denote data that differ significantly, Tukey (*p* < 0.05). PGF2α, Prostaglandin F2alpha; FD, full dose; SD, split dose; SE, Standard Error. * Denotes data that differ significantly.

**Table 5:** Measures of the surface temperature (°C) of lacrimal caruncle of piglets (n = 300) born from gilts induced with PGF2α and treated with caffeine prior to farrowing.

	Control	PGF2α FD	PGF2α SD	PGF2α FD + Caffeine	PGF2α SD + Caffeine	*p*-Value
	Mean ± SE	Mean ± SE	Mean ± SE	Mean ± SE	Mean ± SE	
At birth	34.84 ± 0.24 B	36.18 ± 0.22 A	34.44 ± 0.20 B	34.21 ± 0.32 B	34.40 ± 0.31 B	<0.0001 *
After handling	31.61 ± 0.25 B	34.03 ± 0.28 A	29.37 ± 0.21 D	30.74 ± 0.32 BC	30.21 ± 0.31 CD	<0.0001 *
Colostrated	32.95 ± 0.35 AB	34.27 ± 0.42 A	30.58 ± 0.29 C	31.55 ± 0.51 C	30.97 ± 0.42 C	<0.0001 *
24 h after birth	34.69 ± 0.18 CD	36.27 ± 0.21 A	34.19 ± 0.16 D	35.15 ± 0.28 BC	35.60 ± 0.23 B	<0.0001 *

A, B, C, D Different superscript letters within a row denote data that differ significantly, Tukey (*p* < 0.05). PGF2α, Prostaglandin F2alpha; FD, full dose; SD, split dose; SE, Standard Error. * Denotes data that differ significantly.

**Table 6:** Measures of the surface temperature (°C) of the base of the ear of piglets (n = 300) born from primiparous sows induced with PGF2α and treated with caffeine prior to farrowing.

	Control	PGF2α FD	PGF2α SD	PGF2α FD + Caffeine	PGF2α SD + Caffeine	*p*-Value
	Mean ± SE	Mean ± SE	Mean ± SE	Mean ± SE	Mean ± SE	
At birth	34.73 ± 0.29 B	36.39 ± 0.32 A	34.44 ± 0.25 B	34.46 ± 0.40 B	34.11 ± 0.37 B	<0.0001 *
After handling	31.23 ± 0.29 B	33.40 ± 0.33 A	29.16 ± 0.25 C	29.26 ± 0.39 C	28.55 ± 0.38 C	<0.0001 *
Colostrated	33.47 ± 0.37 A	34.56 ± 0.44 A	30.85 ± 0.30 B	31.51 ± 0.54 B	30.87 ± 0.45 B	<0.0001 *
24 h after birth	36.38 ± 0.17 B	36.80 ± 0.20 AB	36.28 ± 0.15 B	36.61 ± 0.27 AB	37.20 ± 0.22 A	0.0115 *

A, B, C Different superscript letters within a row denote data that differ significantly, Tukey (*p* < 0.05). PGF2α, Prostaglandin F2alpha; FD, full dose; SD, split dose; SE, Standard Error. * Denotes data that differ significantly.

**Table 7:** Weight (g) at 0 and 21 days of age of piglets born from gilts induced with PGF2α and treated with caffeine prior to farrowing.

	Control	PGF2α FD	PGF2α SD	PGF2α FD + Caffeine	PGF2α SD + Caffeine	*p*-Value
	Mean ± SE	Mean ± SE	Mean ± SE	Mean ± SE	Mean ± SE	
Weight 0 d	1318.00 ± 36.41 B	1381.63 ± 36.78 AB	1315.46 ± 26.14 B	1491.03 ± 41.23 A	1406.10 ± 40.21 AB	0.0038 *
Weight 21 d	6133.67 ± 87.81 BC	5929.38 ± 88.72 C	6120.37 ± 68.30 BC	6357.89 ± 99.71 AB	6591.07 ± 116.16 A	<0.0001 *

A, B, C Different superscript letters within a row denote data that differ significantly, Tukey (*p* < 0.05). PGF2α, Prostaglandin F2alpha; FD, full dose; SD, split dose; SE, Standard Error. * Denotes data that differ significantly.

## Data Availability

Data are contained within the article.

## Abbreviations

The following abbreviations are used in this manuscript:

PGF2α	Prostaglandin F2alpha
FD	Full dose
SD	Split dose
